# The chronic effects of a combination of herbal extracts (Euphytose^®^) on psychological mood state and response to a laboratory stressor: A randomised, placebo-controlled, double blind study in healthy humans

**DOI:** 10.1177/02698811221112933

**Published:** 2022-07-23

**Authors:** Fiona Dodd, David Kennedy, Emma Wightman, Julie Khan, Michael Patan, Rian Elcoate, Philippa Jackson

**Affiliations:** 1 Department of Psychology, Brain, Performance and Nutrition Research Centre, Northumbria University, Newcastle upon Tyne, UK; 2 Nutrition Trials at Northumbria (NUTRAN) Northumbria University, Newcastle upon Tyne, UK

**Keywords:** Herbal extract, valerian, passionflower, hawthorn, ballota, stress, anxiety, mood, cognition

## Abstract

**Background:**

Global lifetime prevalence of anxiety disorders has been estimated at approximately 16.6%, with subclinical prevalence likely much higher. Herbal approaches to reduce anxiety may be as effective as pharmacological treatments and are less likely to be associated with adverse side effects. The herbal species, namely, valerian, passionflower, hawthorn and ballota, have a long history of use as anxiolytics in traditional medicine, further supported by recent pre-clinical and clinical trials.

**Aims:**

To assess the effects of chronic (14 days) supplementation with a multi-herb extract preparation (MHEP, Euphytose^®^) on psychological state and psychological and physiological stress responses during a laboratory stressor.

**Methods:**

In this crossover study, 31 healthy participants (aged 19–58 years) received a MHEP and placebo for 14 days with a 28-day washout. Anxiety (State-Trait Anxiety Inventory), mood and physiological measures of stress (heart rate, galvanic skin response, salivary α-amylase and cortisol levels) were measured before and after an Observed Multitasking Stressor. Cognitive performance was also assessed.

**Results:**

MHEP was associated with reduced tension-anxiety (*p* = 0.038), with participants showing an attenuated response to the observed multitasking psychosocial stressor following MHEP, evidenced by lower salivary α-amylase (*p* = 0.041) and galvanic skin response (*p* = 0.004).

**Conclusions:**

The combination of herbal extracts contained within the MHEP reduced subjective anxiety in a healthy population and lowered electrodermal skin conductance and concentration of salivary α-amylase in response to a psychosocial stressor, compared to placebo. The study was registered on clinicaltrials.gov (identifier: NCT03909906).

## Introduction

The global lifetime prevalence of anxiety disorders has been estimated at approximately 16.6% (Remes et al., 2016), with 8.1% of individuals within the United Kingdom having reported suffering from an anxiety disorder including generalised anxiety disorder (GAD), obsessive-compulsive disorder, panic disorder and phobias (McManus et al., 2016). Worryingly, subclinical prevalence is likely much higher (Haller et al., 2014), with substantial increases in GAD observed in younger people in recent years (Slee et al., 2021). Of those that reported suffering from anxiety, 49.9% reported also seeking treatment, 44% of which reported taking medication (McManus et al., 2016). Importantly, mental health in non-clinical populations can be affected by stressors and hassles encountered in daily life. For example, daily hassles in college student populations are shown to be significantly related to anxiety and depression (D’Angelo and Wiekzbicki, 2003), and individual differences in reactivity to daily stressors can predict depressive symptoms (Parrish et al., 2011). Furthermore, stressful and adverse life events have been shown to play a role in the onset of anxiety and depressive symptoms (Zou et al., 2018) as well as anxiety disorders (Miloyan et al., 2018). Therefore, appropriately managing daily stress may be important for long-term mental health and for the prevention of mood disorders.

Herbal approaches to reduce anxiety may be as effective as pharmacological treatments (Andreatini et al., 2002; Murphy et al., 2010) and are less likely to be associated with adverse side effects (Alramadhan et al., 2012; Savage et al., 2018). Several herbal species including *Valeriana officinalis* (valerian), *Passiflora incarnata* L. (passionflower) and *Ballota nigra* L. (ballota) have a long history of use as anxiolytics in traditional medicine (Dhawan et al., 2001a; Shinjyo et al., 2020), further supported by recent pre-clinical and clinical trials. For example, in vitro studies suggest that certain constituents of valerian can bind to and influence the activity of gamma-aminobutyric acid (GABA)_A_ sites (Benke et al., 2009), the same sites influenced by benzodiazepines commonly used as prescribed anxiolytics. Valerian extract has also been found to influence the transport of GABA itself (Santos et al., 1994). While modulation of GABA receptors is thought to be one of the leading mechanisms of action of the plant (Orhan, 2021), the extract has also demonstrated partial agonist activity at serotonin receptors (Dietz et al., 2005) as well as adenosine A_1_ receptor signalling (Shinjyo et al., 2020). In vivo, valerian has potent anxiolytic effects in rodents, with those administered valerian root extract showing significantly lower levels of anxiety than those administered a control substance (Murphy et al., 2010). Valerian, in combination with *Melissa officinalis* (lemon balm), led to significantly lower levels of anxiety during laboratory-induced stress in humans. Here, individuals given a 600-mg dose reported significantly lower levels of anxiety than those given placebo or a higher 1800 mg dose (Kennedy et al., 2006). A similar dose in isolation (530 mg) significantly reduced state anxiety (as measure by the State-Trait Anxiety Inventory (STAI)) following 1 month’s supplementation in haemodialysis patients (Tammadon et al., 2021). Anxiolytic effects have also been demonstrated following lower doses. Individuals administered with a 100-mg dose of valerian within a clinical setting, reported feeling subjectively calmer and less anxious compared to controls when receiving dental surgery (Pinheiro et al., 2014). Similarly, 100 mg of valerian provided comfort and relaxation (in the absence of sedating effects) during molar extraction in anxious patients (Farah et al., 2019). Valerian has also led to increases in frontal alpha activity as measured by electroencephalogram (EEG) following a 300-mg daily dose for 1 month, a finding correlated with anxiolysis (Roh et al., 2019).

*P. incarnata* (passionflower) is an herbal substance that has been seen to provide similar anxiolytic properties as the commonly prescribed benzodiazepine midazolam within dental patients, at a dose of 260 mg (Dantas et al., 2017) and 500 mg (da Cunha et al., 2021). Drops of the extract (equivalent to approximately 500–600 mg) also led to reduced anxiety in patients prior to undergoing periodontal treatment (Kaviani et al., 2013). Additionally, 500 mg passionflower significantly reduced the levels of subjective anxiety when compared to controls in individuals receiving surgery (Movafegh et al., 2008), with similar results found in individuals who underwent spinal anaesthesia following 700 mg passionflower (Aslanargun et al., 2012). Following chronic administration, passionflower has shown similar anxiolytic potency to oxazepam (Akhondzadeh et al., 2001). Within the few studies that investigate the anxiolytic mechanisms of action of passionflower, research has found that passionflower (*Passiflora caerulea*) acts as a partial agonist on benzodiazepine receptors (Appel et al., 2011; Wolfman et al., 1994). Similarly, *B. nigra* (ballota) contains several phenylpropanoids, precursors to flavonoids, which are compounds able to bind to benzodiazepine, dopaminergic and opioid receptors in rodents, possibly explaining the neuro-sedative properties of the plant (Daels-Rakotoarison et al., 2000). Likewise, *Crataegus* sp. (hawthorn) are a species rich in polyphenols including flavonoids and procyanidins. Hawthorn preparations are effective in the treatment of cardiovascular and ischemic heart disease, with hypotensive effects often reported (Tassell et al., 2010). A small pilot study (*N* = 36) assessing the effects of 10 weeks’ administration of 500 mg hawthorn extract alone or in combination with magnesium in mildly hypertensive adults has provided initial evidence of the anxiolytic effects of this extract. Trends for reduced blood pressure and reduced anxiety in those administered the hawthorn extract were observed, both with hawthorn extract alone and in combination with magnesium (Walker et al., 2002).

The multi-herb extract preparation (MHEP), Euphytose^®^, contains extracts of the four aforementioned herbal plants, albeit in smaller doses (50 mg *V. officinalis* L. (from the roots), 40 mg *P. incarnate* L. (aerial parts), 10 mg *Crataegus* sp. (from the leaf and flower) and 10 mg *B. nigra* L. (from the flowering tops)). Evidence has shown that this MHEP combination is able to interact with benzodiazepine receptors, which may underpin the anxiolytic effects (Valli et al., 1991). In outpatients with adjustment disorder and anxious mood, Euphytose plus *Cola nitida* and *Paullinia cupana* has previously reduced scores on the Hamilton Anxiety Rating Scale, compared to placebo, after 28 days’ treatment (Bourin et al., 1997). Currently, evidence to suggest that this specific MHEP is an effective anxiolytic in healthy, sub-clinical populations does not exist within the literature. With the high prevalence of sub-clinical GAD within the general population (Haller et al., 2014), the potential anxiolytic benefits of MHEPs present significant scope for use within this population and warrant further investigation with randomised controlled trials. Previous research has shown that moderate physiological and psychological anxiety and stress responses can be effectively induced in a laboratory context. The Observed Multitasking Stressor (OMS) requires participants to engage with a computerised tracking task and to conduct verbal arithmetic while being monitored by a panel of two researchers. The OMS has been shown previously to invoke a physiological and a psychological stress response, demonstrated by an increase in levels of subjective anxiety as measured by the use of the STAI-State subscale, a validated, widely used measure for fluctuating levels of anxiety (Kennedy et al., 2020; Jackson et al., 2020).

Therefore, the aim of the present study was to assess the effects of chronic (14 days) supplementation with a MHEP (Euphytose) on psychological state with regards to perceived stress and overall mood as well as psychological and physiological stress responses during a laboratory stressor in a sample of healthy, sub-clinical participants.

## Methods

### Study design

A randomised, placebo-controlled, double-blind, crossover design was utilised. Participants attended the Brain, Performance and Nutrition Research Centre laboratory at Northumbria University and were assessed after 14 days supplementation with MHEP and a matched placebo. The study was performed in accordance with the ethical principles that have their origin in the Declaration of Helsinki (1996). The trial was conducted in compliance with protocol/GCP/applicable regulatory requirements and commenced only when a favourable ethical opinion was obtained from the University of Northumbria Department of Psychology Ethics Committee, United Kingdom, approval number 13339.

### Determination of sample size

The power calculation was made with reference to the medium effect size (Cohen’s *d* = 0.56) reported in the study by Meier et al. (2018) for the effect of a combination product containing valerian, passion flower and lemon balm on anxiety as measured using the STAI-State subscale, administered before and at several time points after a psychological stressor. Therefore, with a mixed design study involving the within-subjects factors of treatment and assessment and the between-subjects factor of treatment order on the primary outcome measure (state anxiety-STAI), a total sample size of 28 participants was required to meet the conventionally accepted 80% power to detect a significant difference (α = 0.05) between treatments.

### Study population

A total of 31 healthy adults were randomised, of which 1 withdrew and 3 participants were withdrawn due to major protocol violations, as they did not fully engage with the tasks (identified in each case by numerous statistical outliers and deviations). The remaining 27 participants (19 female), aged 19–58 years (mean = 33.74, SD = 11.19), self-reported being in good health and were free from any relevant medical condition or disease including psychiatric and neurodevelopmental disorders. Blood pressure was taken at screening, and participants were enrolled into the study if it measured <159 mmHg systolic and <99 mmHg diastolic. Participants confirmed they were not currently taking any relevant pharmaceuticals and had not taken any antibiotics within 4 weeks of screening. They also confirmed they had not taken part in another clinical trial within 30 days and had not experienced an event (personal or professional) likely to have impacted their emotional and/or psychological state within the week prior to starting the study and that they did not have an event planned (personal or professional) likely to affect their emotional, psychological or hormonal state during the course of the study. A full list of the inclusion and exclusion criteria can be found in Supplemental File 1. Written informed consent was obtained from participants prior to any research-related procedures being performed. Participants were recruited via an opportunity sample from Northumbria University students and staff and the general population.

### Treatment

Participants received MHEP (dose per tablet; 50 mg *V. officinalis* L., 40 mg *P. incarnate* L., 10 mg *Crataegus* sp. and 10 mg *B. nigra L.*) and a matched placebo in a counterbalanced order. The full composition of the active treatment and placebo is listed in Supplemental File 2. Treatments were delivered from the manufacturer (Bayer HealthCare, Basel, Switzerland) in boxes labelled as placebo and verum. The bottles for each treatment arm were identical. An independent third party who had no further involvement with the trial procedures created a fully counterbalanced computer-generated randomisation schedule (www.randomization.com) and assigned the treatment codes A and B to the treatments. Bottles were labelled with a randomisation number according to the counterbalancing schedule by the lead researcher; randomisation numbers were issued to participants sequentially at visit 1.

**Figure 1.**
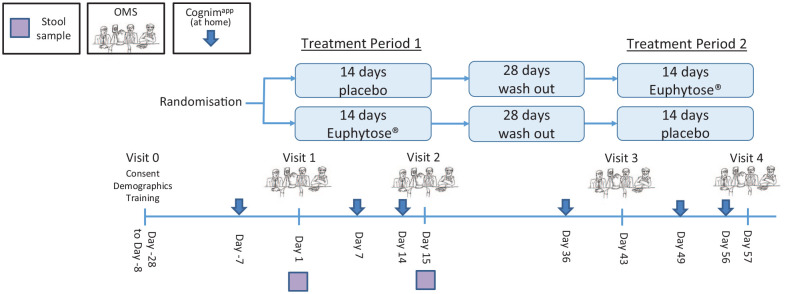
Timeline of study incorporating mood and OMS assessment.

Participants were directed to take two tablets with breakfast, lunch and dinner for a period of 14 days. This was followed by a 28-day washout period, before participants commenced their second treatment period (see Figure 1 for visual representation of treatment schedule). Compliance was assessed at testing visits 2 and 4 by treatment counts and treatment diaries.

### Psychological measures

#### State-Trait Anxiety Inventory

The STAI-‘State’ subscale is a widely used instrument for measuring fluctuating levels of anxiety. The subscale contains 20 statements (e.g. ‘I am calm’) each with a four-point Likert-type scale. Participants rate how much they feel like each statement at the time of making the response. Scores on the STAI range from 20 to 80, with higher scores representing higher levels of anxiety. The Trait subscale also consists of 20 statements but refers to how participants generally feel (Speilberger et al., 1969). STAI State was the primary outcome measure.

#### General Health Questionnaire (GHQ-12)

The GHQ-12 is a screening instrument used for assessing general psychological health in both clinical settings and non-clinical research settings requiring repeated measurements over time. The GHQ-12 consists of 12 items, each assessing the severity of a mental problem over the past few weeks using a four-point scale (0–3) with higher scores indicating worse conditions (Goldberg and Williams, 1988).

#### Perceived stress scale (PSS)

The PSS is a 10-item questionnaire that assesses the degree to which situations in one’s life are appraised as stressful using a five-point scale (0–4). It is a widely used research instrument, and its validity has been established within a number of populations (Froelicher et al., 2004; Golden-Kreutz et al., 2004; Mimura and Griffiths, 2004).

#### Profile of mood states (POMS)

The POMS is a well-established, factor-analytically derived measure of psychological distress for which high levels of reliability and validity have been documented (Heuchert and McNair, 2012). The POMS consists of 65 adjectives rated on a 0–4 scale that can be consolidated into depression-dejection, tension-anxiety, anger-hostility, confusion-bewilderment, vigour-activity and fatigue-inertia subscales. The latter two subscales can be interpreted as measures of fatigue and have been validated as separate factors in a number of studies. Norms have been published for a variety of patient and non-patient groups.

#### Visual analogue mood scales (VAMS)

Participants completed a series of VAMS anchored by 27 antonyms relating to mood and psychological state. Participants moved a marker along the line to describe how they currently feel. Each line was scored as % along the line towards the more positive antonym. Factor analysis of the original 27 items revealed three factors incorporating 18 items (unpublished data). The factors were labelled Alertness (11 items: alert, inattentive; lethargic, energetic; clumsy, coordinated; lively, sluggish; quick-witted, slow-witted; sharp, dull; exhausted, refreshed; bored, engaged; focused, unfocused; drowsy, awake and motivated, unmotivated), Stress (4 items: tense, relaxed; fearful, fearless; stressed, carefree and peaceful, troubled) and Tranquillity (3 items: tranquil, agitated; contented, discontented and friendly, hostile).

#### Observed Multitasking Stressor

**Figure 2.**
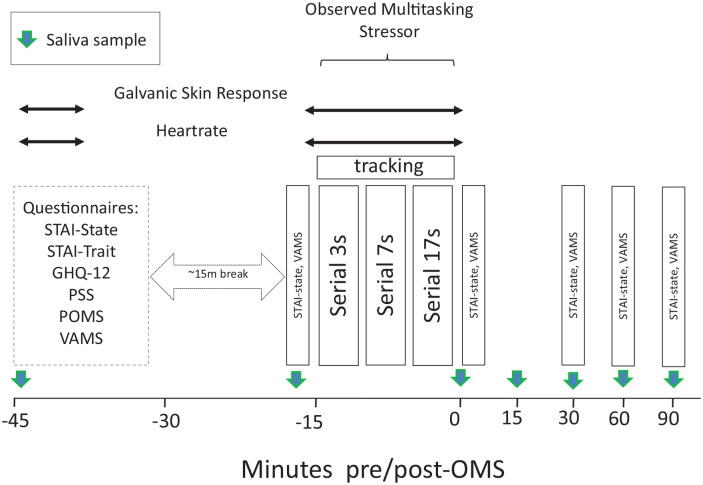
Mood and OMS assessment across study day.

The OMS incorporates two elements that have previously been shown to engender a stress response in laboratory studies; extended multitasking and social evaluation. The OMS has previously been shown to provoke a psychological stress response across repeated administrations (Kennedy et al., 2020). Briefly, the OMS comprised verbal completion of three serial subtraction tasks (3s, 7s and 17s) for 4 min each (12 min in total). Participants were instructed to count backwards from a given, randomly generated, number between 800 and 999 aloud, as quickly as possible. Performance of the task was scored for the total number of correct and incorrect subtractions. In the case of incorrect responses, subsequent responses were scored as correct if they were correct in relation to the new number. During the serial subtraction tasks, participants also completed a computerised tracking task, in which they were required to use the mouse to move a cursor to attempt to track an asterisk that followed a smooth, random, on-screen path; participants were instructed to keep the cursor as close to the asterisk as possible. These tasks were performed in a separate ‘interview’ room, in front of a panel of three ‘judges’ who maintained a neutral demeanour throughout the assessment. The computer screen, showing the tracking task, was projected onto a screen to give the impression that the panel was closely monitoring progress. In the laboratory, before entering the interview room and once back in the laboratory after completing the OMS, mood was assessed with the STAI (state) and computer delivered VAMS indicating the participants’ current level of stress, anxiety, relaxation and calmness (see above). These measures of mood were also repeated every 30 min after completion of the stressor, up to 90 min post-stressor, as shown in Figure 2. A full description of the OMS can be found in Supplemental File 3.

### Physiological measures

#### Heart rate (HR) and galvanic skin response (GSR)

HR and GSR was measured throughout performance of the OMS. GSR and HR were measured on testing visits using the Vilistus Digital Sampling Unit (Durham Systems Management Limited, Penrith, UK). The GSR sensors, which measured relative changes in skin conductance, were attached to the middle and fourth fingertips on the participant’s non-dominant hand using Velcro straps. The HR sensor clip, which measured blood volume pulse (BVP), was placed on the tip of the index finger or thumb on the non-dominant hand. These sensors were attached at least 1 min prior to the commencement of recording to allow for stabilisation of the readings. The unit measured 32 and 128 samples per second for GSR and HR, respectively.

#### Salivary cortisol and salivary α-amylase

Saliva samples were obtained throughout the protocol at various time points (baseline; pre-OMS; post-OMS and 15, 30, 60 and 90 min post-OMS) using salivettes to measure salivary cortisol response (Poll et al., 2007) and salivary α-amylase response (Justino et al., 2017) (Sarstedt Ltd, Numbrecht, Germany). Once collected, samples were spun down at 1000*g* for 2 min. Samples were transferred into Eppendorfs and frozen at −80°C. Before assaying, the samples were thawed and the cortisol and α-amylase levels in the saliva samples were measured using enzyme-linked immunoassay (Salimetrics Ltd, Carlsbad, CA, USA).

### Cognim^app^ smartphone measures

Cognim^app^ (www.cognimapp.com) allows for at home assessment of participants on a range of cognitive and mood measures throughout the course of the intervention period. To capture response to treatment for both morning sleep inertia and ‘post lunch dip’ periods of the day, as well as ongoing effects of treatment on subjective stress and any potential sedative effects of the intervention, the Cognim^app^ assessment (15 min in total) was completed before breakfast and after lunch. A pretreatment Cognim^app^ assessment took place on days 7 and 36 and then again on days 7 and 14 of each treatment period (i.e. days 7, 14, 49 and 56; see Figure 1). Full descriptions of all cognitive tasks are provided in Supplemental File 4.

### Procedure

Participants attended the Brain, Performance and Nutrition Research Centre laboratory (Northumbria University, UK) on five separate occasions. The first was an introductory visit where informed written consent was obtained. Following the introductory visit, participants attended the laboratory at a prearranged time in the afternoon on four separate occasions (visits 1–4). The first and third visits comprised the baseline assessments. Visits 2 and 4 were chronic assessments and occurred 15 days (±3 days) after visits 1 and 3, respectively. Each visit was identical, except for the intervention consumed between visits 1 and 2 and visits 3 and 4 (see Figure 1 for a schematic depicting the timeline of the study).

Upon arrival at visits 1–4, participants were screened for continued eligibility and provided 5-min baseline GSR and HR readings and a baseline saliva sample. Questionnaires were completed to assess psychological mood/state. After a short (approximately 15 min) break, participants were taken to an ‘interview’ room where they underwent the OMS for 15 min in front of a panel of two observers while also being video recorded and having their GSR and HR readings measured throughout. The STAI-State and VAMS were completed in the laboratory immediately prior to and after the OMS and at 30, 60 and 90 min post-OMS. Seven saliva samples were collected in total (see Figure 2 for a schematic depicting the procedure during testing visits 1–4).

Before leaving on testing visits 1 and 3, participants were provided with their treatment. Participants were also instructed to complete the Cognim^app^ assessment battery just before breakfast and after lunch on days 7 and 14 in each treatment period following their baseline Cognim^app^ assessments on days 7 and 36 (see Figure 1 for schematic depicting the study timeline, which also comprises the Cognim^app^ assessments). A full description of the procedure is provided in Supplemental File 5.

### Statistics

For the data collected during the study visits, the general statistical approach comprised the analysis of data collected following each treatment period (i.e. visits 2 and 4), including data collected at the pre-intervention assessment (i.e. visits 1 and 3) as a covariate. The MIXED procedure in SPSS (version 26.0; IBM corp., Armonk, NY, USA) was used for all analyses. For each model, restricted maximum likelihood estimation methods were used and covariance matrix structure was chosen based on the structure that produced the lowest Schwarz’s Bayesian criterion, an indication of the best fitting model (Drton and Plummer, 2017). Subject was included as a random factor where appropriate. Sidak adjustments were made for multiple comparisons where appropriate. To interrogate the chronic effects of treatment irrespective of the OMS stressor, data collected on arrival at the laboratory, −45 min prior to completing the OMS, were analysed including treatment as a fixed factor and pre-intervention values as a covariate. Outcomes included those derived from the POMS, GHQ, PSS, STAI-Trait, STAI-State and VAMS, as well as GSR, BVP and salivary cortisol and salivary α-amylase.

To investigate the effect of treatment on the direct psychological and physiological response to the OMS, data collected at all other time points during the testing visit were analysed in a separate analysis. Outcomes included STAI-State, VAMS, GSR, BVP and salivary cortisol and salivary α-amylase. These were analysed as above, including treatment and assessment as fixed factors and pre-intervention values as a covariate. For the dual tasking performance outcomes, task was included as an additional factor.

The Cognim^app^ data were analysed as above, including the fixed factors treatment, visit and time of day.

In order to assess the stress response elicited by the OMS procedure itself, the VAMS mood, STAI-State and saliva analyte outcomes collected at visits 1 and 3 in the absence of treatment were analysed as above including assessment and visit as fixed factors.

Missing data were left as empty cells as the linear mixed model approach that was applied to the data uses maximum likelihood to estimate the missing values.

## Results

**Figure 3.**
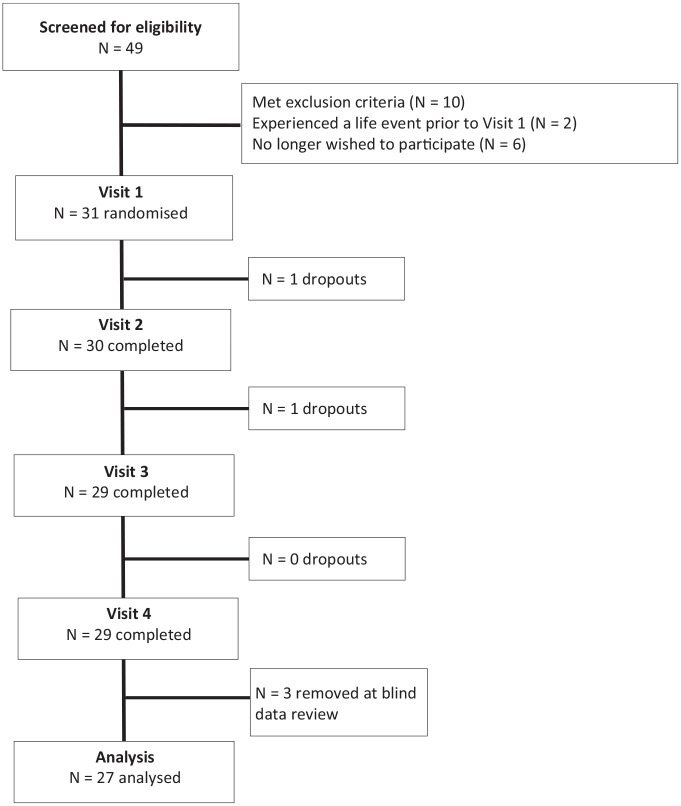
Flow diagram of disposition of subjects throughout the study.

Thirty-one participants were randomised to receive treatment (see Figure 3). One participant withdrew post-randomisation following testing visit 1 and one following testing visit 2 (this data was included in the analysis). Please see Supplemental Tables for data from all measures.

### Handling of missing data

One participant only completed the first phase of the trial including visits 1 and 2. However, these data were included in the analysis; therefore, 27 data sets were eligible for analysis.

### Demographic and other baseline characteristics

Participant demographics and baseline characteristics are summarised in Table 1 below.

**Table 1.** Baseline characteristics (*N* = 27).

Measure	Mean	SD
Sex ratio (male/female)	0.42	
Age (years)	33.74	11.19
Race (frequency *N*)		
White	21	
Asian	3	
Black	1	
Mixed race	2	
Education (years)	17.52	2.78
Dietary restrictions (frequency *N*)		
None	22	
Vegetarian	1	
Vegan	1	
Pescetarian	3	
Fruit and veg consumption (portions/day)	4.02	1.66
Alcohol consumption (units/day)	0.66	0.74
Caffeine consumption (mg/day)	187.30	106.62
Systolic BP (mmHg)	118.31	10.86
Diastolic BP (mmHg)	78.42	7.06
Heart rate (beats/min)	73.43	9.49
BMI (kg/m^2^)	24.70	3.49

BMI: body mass index; BP: blood pressure; SD: standard deviation.

### Compliance and treatment guessing

Compliance was at 97.2% during the placebo phase and 98.3% during the MHEP phase of the study. Compliance was based on (returned) treatment counts. Participants responses to the treatment guess questionnaire, completed on the final visit, were analysed via a chi-square test and revealed that there was no significant difference between the ability to correctly detect the active treatment and the placebo (χ^2^(1) = 0.619, *p* = 0.431).

### Baseline comparisons

Pre-intervention visit data (i.e. visits 1 and 3) were analysed for treatment group effects and treatment group × visit interactions to confirm an absence of baseline differences between the groups, or carryover effects from the first treatment period.

No baseline differences were observed for any of the outcomes included in the chronic effects analysis or any of the Cognim^app^ outcomes.

With regards to the analysis of OMS-associated effects for data that were collected between −15 min pre-OMS until 90 min post-OMS, a significant effect of treatment group was observed for state anxiety (*F*(1, 222.98) = 8.43, *p* = 0.004). Participants assigned to MHEP reported lower anxiety (30.81) than placebo (32.97) before treatment commenced. A significant effect of treatment was also observed for the OMS dual task speed (*F*(1, 282.1) = 7.30, *p* = 0.007) and accuracy (*F*(1, 280.3) = 14.79, *p* < 0.001) measures (*z* scores). Participants assigned to MHEP were faster (0.14) and more accurate (0.18) than those assigned to placebo (−0.12 and −0.20, respectively) before treatment commenced.

### Effect of the OMS (in the absence of treatment)

**Figure 4.**
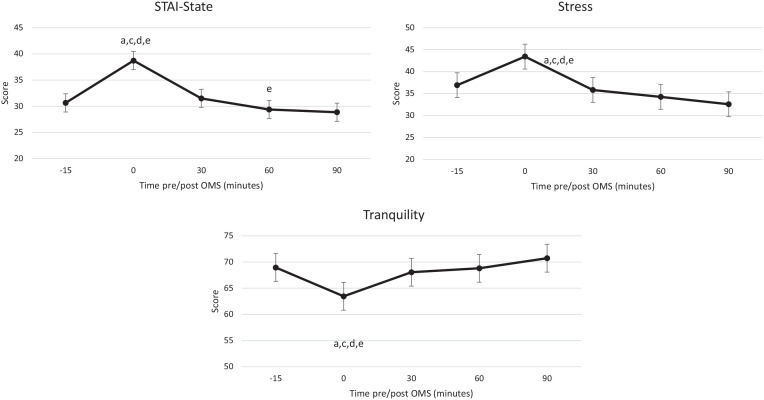
Estimated marginal means for state anxiety (top), stress (middle) and tranquillity (bottom). State anxiety was derived from the STAI, and stress and tranquillity were derived from VAMS. Data collected at visits 1 and 3 are presented by assessment time across the study visit. Small letters indicate significant (*p* < 0.05) post hoc comparisons: a, −15 min; c, 30 min; d, 60 min; e, 90 min. OMS: Observed Multitasking Stressor.

A significant effect of assessment was observed for state anxiety (*F*(4, 167.91) = 38.71, *p* < 0.001), stress (*F*(4, 229.06) = 13.99, *p* < 0.001) and tranquillity (*F*(4, 229.05) = 8.14, *p* < 0.001). Post hoc comparisons revealed that the assessment completed immediately after the OMS was significantly higher (state anxiety, stress) or lower (tranquillity) compared to all the other assessments (Figure 4).

An effect of visit was detected for state anxiety (*F*(1, 55.33) = 9.26, *p* = 0.004), with lower anxiety reported at visit 3 (30.24) compared to visit 1 (33.40). Similarly, an effect of visit was also observed for alertness (*F*(1, 229.38) = 9.15, *p* = 0.003), with higher alertness reported at visit 3 (65.08) compared to visit 1 (62.64).

Together these findings indicate that completion of the OMS had the anticipated effect on psychological mood state. The effect of visit suggests mild habituation to the protocol, but this did not interact with assessment on any of the outcomes.

**Figure 5.**
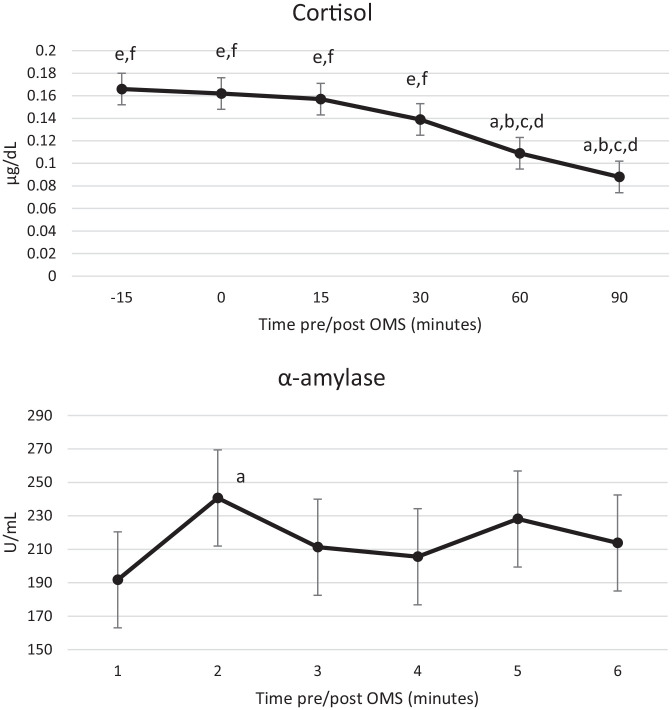
Estimated marginal means for salivary cortisol (top) and salivary α-amylase (bottom). Data collected at visits 1 and 3 are presented by assessment time across the study visit. Small letters indicate significant (*p* < 0.05) post hoc comparisons: a, −15 min; b, 0 min; c, 15 min; d, 30 min; e, 60 min; f, 90 min. OMS: Observed Multitasking Stressor.

A significant effect of assessment was also observed for salivary α-amylase (*F*(5, 202.78) = 3.83, *p* = 0.002). Post hoc comparisons revealed that the value of the sample collected immediately following the OMS (240.69) was significantly higher than the sample collected immediately prior to the OMS (191.74; *p* = 0.003). A significant effect of assessment was also observed for salivary cortisol (*F*(5, 206.18) = 10.29, *p* < 0.001). However, the pattern of response here was more anticipatory; post hoc comparisons revealed that cortisol concentration was elevated from −15 min pre-OMS and only began to decline 60 min post OMS (Figure 5).

### Chronic effects analysis in the presence of treatment (MHEP)

**Figure 6.**
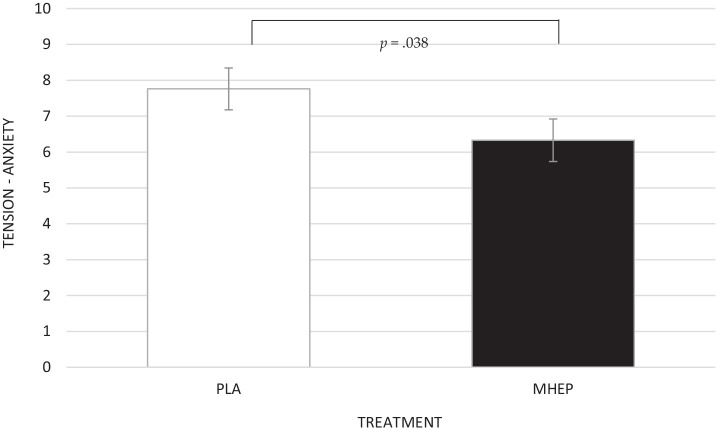
Estimated marginal means and standard errors (±SE) for post intervention tension-anxiety by treatment group. PLA: placebo; MHEP: multi-herb extract preparation.

A significant main effect of treatment was identified for tension-anxiety on the POMS questionnaire (*F*(1, 22.13) = 4.84, *p* = 0.038), with post hoc pairwise comparisons revealing MHEP resulted in significantly lower tension-anxiety (6.33) than placebo (7.75) (Figure 6).

#### Psychological and physiological response to the OMS

##### Salivary cortisol and salivary α-amylase

**Figure 7.**
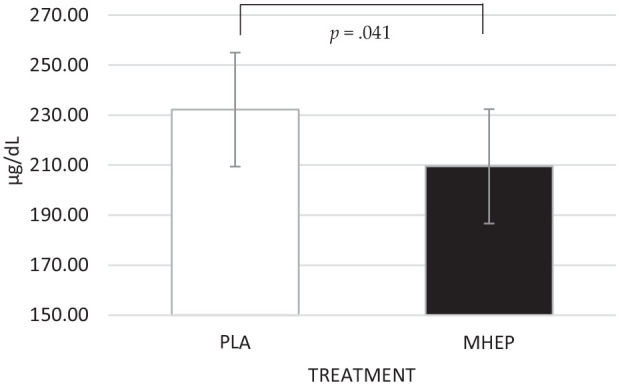
Estimated marginal means and standard errors (±SE) for salivary α-amylase. A treatment effect revealed that α-amylase was significantly lower following MHEP, compared to placebo overall during the OMS assessment. PLA: placebo; MHEP: multi-herb extract preparation.

A significant main effect of treatment was identified for salivary α-amylase (*F*(1, 268.32) = 4.20, *p* = 0.041), with participants having lower salivary α-amylase following MHEP (209.51) compared to placebo (232.21) overall during the OMS assessment (Figure 7).

##### Galvanic skin response

**Figure 8.**
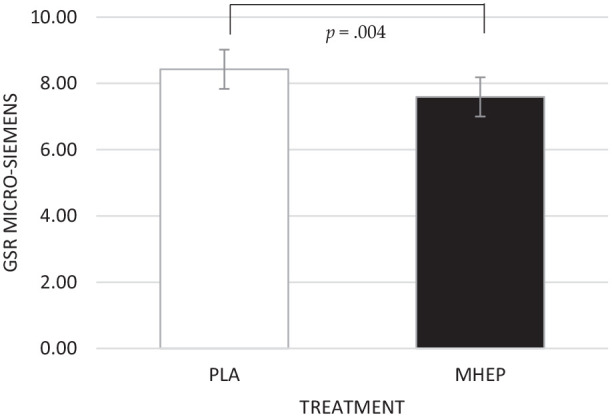
Estimated marginal means and standard errors (±SE) for GSR µSiemens by treatment group. PLA: placebo; MHEP: multi-herb extract preparation.

A significant main effect of treatment was identified for GSR (*F*(1, 119.20) = 8.63, *p* = 0.004), with participants having a lower GSR following MHEP (7.59) than placebo (8.43) overall during the OMS assessment (Figure 8).

##### Cognim^app^ smartphone measures

A significant interaction between treatment × time of day was identified for digit vigilance false alarms (*F*(1, 127.61) = 4.13, *p* = 0.044). However, post hoc pairwise comparisons revealed no significant differences between the groups.

**Figure 9.**
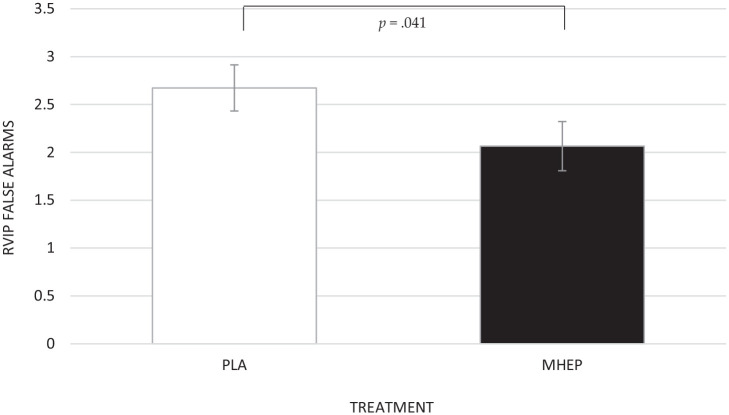
Estimated marginal means and standard errors (±SE) for post intervention RVIP false alarms by treatment group. PLA: placebo; MHEP: multi-herb extract preparation.

A significant main effect of treatment was identified for rapid visual information processing (RVIP) false alarms (*F*(1, 132.86) = 4.27, *p* = 0.041), with post hoc pairwise comparisons revealing that MHEP made significantly less false alarms (2.07) than placebo (2.67) (Figure 9).

**Figure 10.**
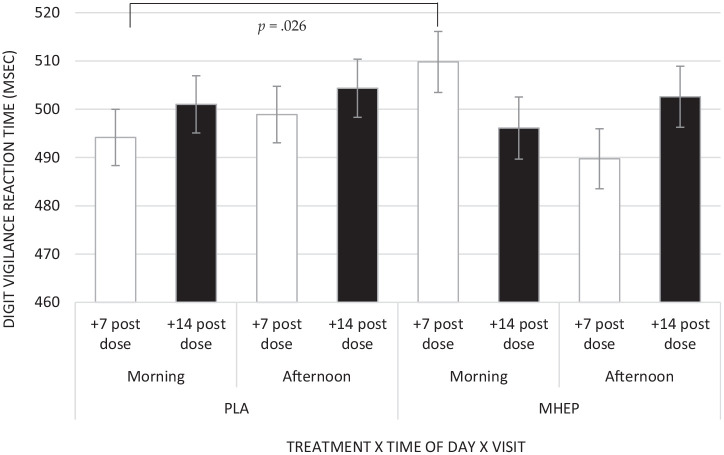
Estimated marginal means and standard errors (±SE) for post intervention digit vigilance reaction time for treatment × time of day × visit. PLA: placebo; MHEP: multi-herb extract preparation.

A significant interaction between treatment × visit × time of day was identified for digit vigilance reaction time (*F*(2, 123.28) = 3.42, *p* = 0.036), with post hoc pairwise comparisons revealing that placebo had significantly faster reaction times (494.16 ms) than MHEP (509.80 ms) but only in the +7 day morning assessment (*p* = 0.026) (Figure 10).

## Discussion

In the current study, 14 days’ supplementation with MHEP was associated with reduced tension-anxiety. In addition, participants showed an attenuated response to the OMS psychosocial stressor following MHEP, evidenced by lower salivary α-amylase and GSR. With regards to cognitive performance assessed at home via Cognim^app^, MHEP led to significantly fewer false alarms on the RVIP task compared with placebo. A significant reduction in speed of performance on the digit vigilance task following MHEP was also observed. However, this isolated negative effect was only observed during the morning assessment on day 7 and appears to contradict the pattern of response for the other assessments where performance was numerically faster following MHEP.

Concerning mood, tension-anxiety was significantly lower following MHEP, compared to placebo. The POMS questionnaire from which this measure is derived was completed prior to the start of the study day and therefore represents a reduction in tension-anxiety following 14 days’ treatment. Of the species contained within the extract, both passionflower and valerian have demonstrated subjective anxiolytic properties within the literature following an acute, sometimes larger, dose of the individual extracts (Aslanargun et al., 2012; Farah et al., 2019; Movafegh et al., 2008; Pinheiro et al., 2014). Since the quantities of valerian and passionflower contained within the MHEP are in some cases lower than those previously observed to have anxiolytic effects, the improvement in subjective anxiety seen here may represent the cumulative effect of a smaller dose of each extract. In terms of mechanisms, the sesquiterpene valerenic acid contained within valerian (when extracted from the underground organs of the *V. officinalis* species as in MHEP) has been shown to increase central GABA levels. This leads to a reduction in central nervous system activity (Houghton, 1999), which may have contributed to the reduction in tension-anxiety observed here following MHEP. It has been demonstrated that the ratio of valerenic acid to acetoxy valerenic acid contained within the extract is of importance in this regard, with extracts containing higher levels of valerenic acid leading to more pronounced anxiolytic effects (Becker et al., 2014; Felgentreff et al., 2012; Trauner et al., 2008). With regards to the *Passiflora* species, despite a long history of use as an anxiolytic, its mechanism of action is not well understood. A role of the flavonoid chrysin in the agonism of benzodiazepine receptors has been proposed (Appel et al., 2011; Wolfman et al., 1994); however, consensus here is lacking (Movafegh et al., 2008). Interestingly, the anxiolytic activity profile of the *P. incarnata* extract is reportedly determined by the parts of the plant used, with the roots shown to be devoid of anxiolytic effects (Dhawan et al., 2001b) and the leaves said to contain maximum concentrations of bioactive constituents (Dhawan et al., 2004). Importantly, the *P. incarnata* extract contained within MHEP is obtained from the aerial parts of the plant. It should be noted that this was an isolated effect on mood, and there was no evidence of a chronic effect of treatment on state or trait anxiety – the primary outcome measure – or any other of the mood measures. This positive effect, albeit in the expected direction, should therefore be interpreted with caution. One consideration here is the context in which the pre-dose mood and well-being questionnaires were administered. Participants completed these questionnaires and mood scales in full knowledge that they were going to complete the OMS, and this may have influenced their responses on these questionnaires. Subjective well-being has been shown to correlate with current mood (Yardley and Rice, 1991) and is also affected by experimental manipulation (Yap et al., 2017). It may be that that anticipation of the OMS masked any chronic effect of treatment on state anxiety or indeed any of the other subjective measures.

With regards to the physiological measures collected during the OMS procedure, an increase in the electrodermal skin conductance response (measured in µSiemens) is recognised as a good indicator of activation of the sympathetic nervous system (Dawson et al., 2017). The observed attenuation of this response during performance of the OMS following MHEP compared to placebo is therefore an indicative of a beneficial effect of the treatment. Similarly, a reduction in salivary α-amylase was also observed across the study day following MHEP. Salivary α-amylase is considered a valid measure of autonomic nervous system (ANS) activation (Nater and Rohleder, 2009), a reduction of which would also indicate an attenuation of the stress response. Euphytose has been shown to interact with benzodiazepine receptors, which has been proposed as the potential mechanism for its anxiolytic effects (Valli et al., 1991). Previously valerian has been shown to reduce HR during a mentally stressful cognitive task following 7 days administration (Cropley et al., 2002), a finding not replicated in the present study following 14 days administration. However, this was following a considerably larger dose of 600 mg, as compared to the 300 mg daily dose contained within the MHEP. Similarly, an acute 260 mg dose of passionflower was observed to have the same effect on HR as the drug Midazolam, when administered prior to tooth extraction surgery (Dantas et al., 2017), but, again, this is a larger dose than the 80 mg administered acutley here. Taking into consideration the quantities of each extract contained within MHEP, it is possible that skin conductance and salivary α-amylase are more sensitive to the effects of the lower doses administered here.

Although it could be expected that the active treatment would have a beneficial effect across all the physiological parameters, it should be noted that inconsistencies in these measures are also found in the literature. Cortisol, a steroid hormone, is a reliable measure of the response to acute stress (Hellhammer et al., 2009). A-amylase, an enzyme found in saliva and involved in digestion, is considered to be a good indicator of ANS activation, although debate exists over whether levels obtained during stressful situations represent sympathetic or parasympathetic activity, or a combination of both (Ali and Nater, 2020). It is of note here that where laboratory-induced psychological stress paradigms have been adopted previously (including the Trier Social Stress Test), a correlation of salivary α-amylase and cortisol levels was not observed (Chatterton et al., 1996; Nater et al., 2005), leading to the suggestion that these two measures react as a consequence of different, albeit linked, stress systems (Nater et al., 2005). Furthermore, studies that have compared the α-amylase and cortisol response to behavioural stress-reduction interventions have reported a reduction in α-amylase levels in the absence of a change in cortisol levels (Ali and Nater, 2020). In the present study, analysis of the pre-intervention study visit data showed that cortisol was already elevated at the −15 min pre-OMS time point – indicative of an anticipatory response to the protocol – which may have also contributed to the null effects on this measure. As described above, this anticipatory response to the stressor was also reflected in an absence of findings on the STAI-State subscale following treatment. Although mild habituation to the OMS at day 14 may also provide some explanation for the absence of effects (on cortisol and the state anxiety), previous research has demonstrated that the OMS is capable of provoking a psychological response following repeated administrations even on the same day (Kennedy et al., 2020).

Considering the Cognim^app^ cognitive performance outcomes, MHEP led to significantly fewer false alarms on the RVIP task compared with placebo. However, the findings here do not appear to represent a consistent pattern of effects for either treatment, rendering interpretation difficult. Specifically, digit vigilance reaction time was significantly slower following MHEP compared to placebo in the +7 day morning assessment. The number of dependent variables should also be acknowledged; the small effects seen here may not have been detected if the number of analyses conducted were adjusted for. Importantly, despite these minimal and contradictory effects, the null findings overall provide evidence of an absence of consistent adverse effects on performance observed either during the study visit or on the Cognim^app^ assessments as a result of the active treatment. Furthermore, we also observed no effect of MHEP on subjective alertness or on the KSS, a reliable measure of subjective drowsiness. Of the extracts contained within MHEP, those understood to have sedating properties include valerian and ballota. The ability of valerian to bind to adenosine receptors has been reported within animal studies and proposed as one of the mechanisms by which the sedating effects may occur (Murphy et al., 2010). The flowered aerial parts of the *B. nigra* L. species (also contained within MHEP) have been used traditionally for their sedative properties, among others (Al-Snafi, 2015; Gruenwald et al., 2000). Although there is little evidence within the literature for its efficacy in humans (Morteza-Semnani and Ghanbarimasir, 2019), animal studies have demonstrated the ability of phenylpropanoids within the extract bind to benzodiazepine, dopaminergic and opioid receptors which may explain, in part, its neuro-sedative properties (Daels-Rakotoarison et al., 2000). Therefore, despite the reported sedating effects of some of the extracts contained within the treatment, MHEP was not associated with any changes in subjective arousal or any consistent negative effects on cognitive performance.

A potential limitation of the current design was the timing of the mood questionnaires and their proximity to the OMS. It could be argued that completing the mood questionnaires immediately prior to the OMS would allow interrogation of the effect of MHEP on anticipatory responses to the stressor; however, it is possible that their completion within the laboratory on the same day as the testing visit may have masked any chronic effect of treatment on subjective mood, which is what they were intended to measure. In future, to determine the effect of treatment on general subjective mood (in the absence of an acute stressor), it is recommended that chronic assessments of mood should be completed in a more neutral setting, on a different day to the OMS in order to capture any potentially subtle effects of treatment.

Cognim^app^ is a valuable assessment tool with the ability to capture cognitive performance and mood measures in any setting, but inevitably this comes with some practical limitations. A laboratory setting provides a quiet environment, free from daily distractions where engagement can be monitored by a study team. Although guidance is provided to the participant to complete the Cognim^app^ assessments with these principles in mind, it is not always practicable when fitting the assessments into their daily lives. Without the ability to monitor participants, there is also the possibility that assessments will not be completed within the appropriate timeframe. In order to monitor time of day effects, including the impact of morning sleep inertia and the post-lunch dip, participants were required to complete the assessments before breakfast and 1 h (2 h maximum) after finishing their lunch. It was evident from the raw data that not all participants adhered to this period and/or consumed breakfast and lunch at irregular times of the day. However, it could be argued that ‘real life’ environments provide the ideal setting within which to assess cognitive performance since any findings determined as a result, either positive or negative, would potentially be even more valid. It is likely that a larger data set with this measure would tease out many of these nuances and individual differences to reveal a clearer pattern of effects.

The findings of the present study demonstrate that 14 days’ supplementation with a combination of the herbal extracts valerian, passionflower, ballota and hawthorn reduces subjective anxiety in a healthy population and lowers electrodermal skin conductance and concentration of salivary α-amylase in response to a psychosocial stressor, compared to placebo. Future studies may benefit from conducting mood and well-being assessments in the absence of the OMS to remove any anticipatory effects of this measure and/or assessing all physiological and mood outcomes over a longer pre- and post-OMS time frame in order to ascertain what the extent of the effect of the preparatory response is in this environment.

## Supplemental Material

sj-doc-1-jop-10.1177_02698811221112933 – Supplemental material for The chronic effects of a combination of herbal extracts (Euphytose®) on psychological mood state and response to a laboratory stressor: A randomised, placebo-controlled, double blind study in healthy humans Click here for additional data file. Supplemental material, sj-doc-1-jop-10.1177_02698811221112933 for The chronic effects of a combination of herbal extracts (Euphytose®) on psychological mood state and response to a laboratory stressor: A randomised, placebo-controlled, double blind study in healthy humans by Fiona Dodd, David Kennedy, Emma Wightman, Julie Khan, Michael Patan, Rian Elcoate and Philippa Jackson in Journal of Psychopharmacology

sj-docx-2-jop-10.1177_02698811221112933 – Supplemental material for The chronic effects of a combination of herbal extracts (Euphytose®) on psychological mood state and response to a laboratory stressor: A randomised, placebo-controlled, double blind study in healthy humans Click here for additional data file. Supplemental material, sj-docx-2-jop-10.1177_02698811221112933 for The chronic effects of a combination of herbal extracts (Euphytose®) on psychological mood state and response to a laboratory stressor: A randomised, placebo-controlled, double blind study in healthy humans by Fiona Dodd, David Kennedy, Emma Wightman, Julie Khan, Michael Patan, Rian Elcoate and Philippa Jackson in Journal of Psychopharmacology

sj-docx-3-jop-10.1177_02698811221112933 – Supplemental material for The chronic effects of a combination of herbal extracts (Euphytose®) on psychological mood state and response to a laboratory stressor: A randomised, placebo-controlled, double blind study in healthy humans Click here for additional data file. Supplemental material, sj-docx-3-jop-10.1177_02698811221112933 for The chronic effects of a combination of herbal extracts (Euphytose®) on psychological mood state and response to a laboratory stressor: A randomised, placebo-controlled, double blind study in healthy humans by Fiona Dodd, David Kennedy, Emma Wightman, Julie Khan, Michael Patan, Rian Elcoate and Philippa Jackson in Journal of Psychopharmacology

sj-docx-4-jop-10.1177_02698811221112933 – Supplemental material for The chronic effects of a combination of herbal extracts (Euphytose®) on psychological mood state and response to a laboratory stressor: A randomised, placebo-controlled, double blind study in healthy humans Click here for additional data file. Supplemental material, sj-docx-4-jop-10.1177_02698811221112933 for The chronic effects of a combination of herbal extracts (Euphytose®) on psychological mood state and response to a laboratory stressor: A randomised, placebo-controlled, double blind study in healthy humans by Fiona Dodd, David Kennedy, Emma Wightman, Julie Khan, Michael Patan, Rian Elcoate and Philippa Jackson in Journal of Psychopharmacology

sj-docx-5-jop-10.1177_02698811221112933 – Supplemental material for The chronic effects of a combination of herbal extracts (Euphytose®) on psychological mood state and response to a laboratory stressor: A randomised, placebo-controlled, double blind study in healthy humans Click here for additional data file. Supplemental material, sj-docx-5-jop-10.1177_02698811221112933 for The chronic effects of a combination of herbal extracts (Euphytose®) on psychological mood state and response to a laboratory stressor: A randomised, placebo-controlled, double blind study in healthy humans by Fiona Dodd, David Kennedy, Emma Wightman, Julie Khan, Michael Patan, Rian Elcoate and Philippa Jackson in Journal of Psychopharmacology

sj-docx-6-jop-10.1177_02698811221112933 – Supplemental material for The chronic effects of a combination of herbal extracts (Euphytose®) on psychological mood state and response to a laboratory stressor: A randomised, placebo-controlled, double blind study in healthy humans Click here for additional data file. Supplemental material, sj-docx-6-jop-10.1177_02698811221112933 for The chronic effects of a combination of herbal extracts (Euphytose®) on psychological mood state and response to a laboratory stressor: A randomised, placebo-controlled, double blind study in healthy humans by Fiona Dodd, David Kennedy, Emma Wightman, Julie Khan, Michael Patan, Rian Elcoate and Philippa Jackson in Journal of Psychopharmacology

sj-xlsx-7-jop-10.1177_02698811221112933 – Supplemental material for The chronic effects of a combination of herbal extracts (Euphytose®) on psychological mood state and response to a laboratory stressor: A randomised, placebo-controlled, double blind study in healthy humans Click here for additional data file. Supplemental material, sj-xlsx-7-jop-10.1177_02698811221112933 for The chronic effects of a combination of herbal extracts (Euphytose®) on psychological mood state and response to a laboratory stressor: A randomised, placebo-controlled, double blind study in healthy humans by Fiona Dodd, David Kennedy, Emma Wightman, Julie Khan, Michael Patan, Rian Elcoate and Philippa Jackson in Journal of Psychopharmacology
