# Supplementary material for: The chronic effects of a combination of herbal extracts (Euphytose®) on psychological mood state and response to a laboratory stressor: A randomised, placebo-controlled, double blind study in healthy humans
Source: J Psychopharmacol. 2022 Jul 23;36(11):1243–56. doi: 10.1177/02698811221112933 (PMC9643820; doi:10.1177/02698811221112933)
Supplement: sj-docx-2-jop-10.1177_02698811221112933 – Supplemental material for The chronic effects of a combination of herbal extracts (Euphytose®) on psychological mood state and response to a laboratory stressor: A randomised, placebo-controlled, double blind study in healthy humans [file sj-docx-2-jop-10.1177_02698811221112933.docx]

**Supplemental File 1 - Full list of all inclusion/exclusion criteria**

**Inclusion Criteria**

- Aged 18-65 years
- In good physical health
- Subjects agree to abstain throughout the trial from all dietary supplements (note: protein shakes and garlic supplements are allowed)
- Body Mass Index above 18 kg/m^2^ and below 35 kg/m^2^
- Subjects are, in the opinion of the investigator, willing to participate in all scheduled visits and to adhere to all study procedures
- Subjects accept to refrain from alcohol intake 24 hours prior to Testing Visits 1-4
- Subjects do not have a current diagnosis of a significant medical condition which may interfere with the subject’s ability to perform assessments and successfully completes training
- Subjects provide a personally signed and dated informed consent indicating that the subject has been informed of all pertinent aspects of the trial and understood and accepts these
- Have a bank account (required for payment)

## Exclusion Criteria

- Have any pre-existing medical condition/illness

*NOTE: the exceptions to this are controlled (medicated) arthritis, asthma, high cholesterol and reflux-related conditions but they MUST be recorded in the Medical History section*

*Please note – hay fever sufferers are excluded from this study*

*Others may be considered and permitted if deemed not to interact with the active treatment or impede/affect performance- they should be considered on a case-by-case basis*

- Event (personal or professional) likely to have impacted the subject’s emotional and/ or psychological state within the last 1 week (for example but not restricted to: change of professional function/situation, death of a family member, divorce, surgery, accident, etc.)
- Event (personal or professional) likely to affect the subject’s emotional, psychological or hormonal state planned during the study, including vaccination, important medical exam etc
- Smoking or the use of any nicotine replacement products e.g. vaping, gum, patches (smoking within the last 3 months)
- Blood pressure >159/99mmHg
- Excessive use of caffeine (> 500 mg caffeine per day) from all dietary sources
- Current intake of pharmaceuticals (excluding contraception and arthritis, high cholesterol, reflux and hormone replacement treatments (for female participants) where symptoms are stable and medications do not contraindicate the study outcomes and inhalers used as required). Please note participants who take hay fever medication even ‘as required’ are excluded from this study
- Have taken antibiotics within the last 4 weeks
- Any known active infections
- Have a recent history of (within 12 months of screening visit) or strong potential for alcohol or substance abuse. Alcohol abuse is defined as more than 60g (men) / 40g (women) pure alcohol per day (7 / 5.5 units)
- A history of neurological or psychiatric diseases excluding anxiety or depression
- A diagnosis/treatment for a psychiatric disorder within the past 12 months (including anxiety or depression)
- A history of significant head trauma
- Have sleep disturbances and/or are taking sleep aid medication
- Have learning difficulties or dyslexia
- Have visual impairment that cannot be corrected with glasses or contact lenses (including colour blindness)
- Have frequent migraines that require medication (more than or equal to 1 per month)
- Not proficient in English equivalent to IELTS band 6 or above
- Are pregnant, trying to get pregnant or breast feeding
- Have any health condition that would prevent fulfillment of the study requirements
- Any condition which may interfere with the subjects ability to perform assessments
- Are employed in a job that includes night shift work
- Have no access to the internet (including via smartphone)
- Are unable to perform the computerized tests during training to the established acceptable levels for participation in this type of study
- Have habitually used supplements, within the last month (defined as more than 3 consecutive days or 4 days in total)
- Participation in another clinical trial within 30 days prior to screening
- Any history of hypersensitivity to the investigational product or its active or inactive constituents or any food allergy or intolerance
- Non-compliant in terms of treatment consumption
