# Supplementary material for: The chronic effects of a combination of herbal extracts (Euphytose®) on psychological mood state and response to a laboratory stressor: A randomised, placebo-controlled, double blind study in healthy humans
Source: J Psychopharmacol. 2022 Jul 23;36(11):1243–56. doi: 10.1177/02698811221112933 (PMC9643820; doi:10.1177/02698811221112933)
Supplement: sj-docx-3-jop-10.1177_02698811221112933 – Supplemental material for The chronic effects of a combination of herbal extracts (Euphytose®) on psychological mood state and response to a laboratory stressor: A randomised, placebo-controlled, double blind study in healthy humans [file sj-docx-3-jop-10.1177_02698811221112933.docx]

**Supplemental file 2 – Composition of active treatment (Euphytose®) and placebo**

Table 1. Euphytose® ingredient list

| **Active Ingredients** | **Excipients** |
| --- | --- |
| Valerian hydroalcoholic dry extract | Magnesium oxide medium heavy |
| Passion flower hydroalcoholic dry extract | Maltodextrin |
| Hawthorn aqueous dry extract | Cellulose microcrystalline |
| Black Horehound aqueous dry | Magnesium stearate |
|  | Shellac |
|  | Acacia (Arabic gum) |
|  | Sucrose |
|  | Talc |
|  | Carnauba wax |
|  | Sepisperse AS 5924SP |
|  | (Titanium dioxide and Iron oxides mixture) |

Table 2. Placebo ingredient list

| **Tablet core** | **Tablet coating** |
| --- | --- |
| Maltodextrine glucidex it 19 | Ethanol  96 % |
| Cellulose ph 102 | Gomme laque lemon (shellac lemon) |
| Vegetable magnesium stearate | Talc |
|  | Purified water |
|  | Gum arabic |
|  | Refined sugar |
|  | Sepisperse as 5924 |
|  | Carnauba wax |
