# Supplementary material for: The chronic effects of a combination of herbal extracts (Euphytose®) on psychological mood state and response to a laboratory stressor: A randomised, placebo-controlled, double blind study in healthy humans
Source: J Psychopharmacol. 2022 Jul 23;36(11):1243–56. doi: 10.1177/02698811221112933 (PMC9643820; doi:10.1177/02698811221112933)
Supplement: sj-docx-4-jop-10.1177_02698811221112933 – Supplemental material for The chronic effects of a combination of herbal extracts (Euphytose®) on psychological mood state and response to a laboratory stressor: A randomised, placebo-controlled, double blind study in healthy humans [file sj-docx-4-jop-10.1177_02698811221112933.docx]

**Supplemental file 3 – Description of the Observed Multitasking Stressor (OMS)**

Previous research has shown that moderate physiological and psychological anxiety and stress responses can be effectively induced in a laboratory context by ‘interview’ style stressors, which take place in front of a panel of observers. However, methods such as the Trier Social Stress Test (Kirschbaum et al., 1993) rely on an element of surprise (prior to free speech and mental arithmetic) that makes them unsuitable for repeated applications over time. Computerised multi-tasking stressors (e.g. Kennedy et al., 2004), in which participants perform multiple tasks at once, engender milder stress responses, but these responses are sustained across multiple applications. The OMS combines elements of both of these laboratory stressors and comprises an extended period of multi-tasking (verbal serial subtractions plus a concomitant computerised tracking task) whilst being observed by a panel of two researchers. The session was also recorded using a video camera. This stressor has previously been shown in pilot studies conducted within our laboratory (unpublished data) to provoke a physiological response as assessed by heart rate and galvanic skin response, to increase anxiety as assessed with the STAI-state, to decrease calmness as assessed by Bond-Lader mood scales, and to increase subjective ratings of stress whilst reducing ratings of relaxation. These effects have been observed across multiple assessments on multiple days. The tasks can, if required, be scored to give a performance measure. Alternatively, the stressor can remain unscored, simply being employed in order to engender subjective stress and increased anxiety.

The OMS comprised verbal completion of three serial subtraction tasks (3’s, 7’s and 17’s) for 4 minutes each (12 minutes in total). Participants were instructed to count backwards from a given randomly generated number between 800 and 999 aloud, as quickly as possible. Prior to commencing the task, they were instructed verbally that if they made a mistake they should carry on subtracting from the new incorrect number. Performance of the task was scored for the total number of correct subtractions and incorrect subtractions. In the case of incorrect responses, subsequent responses were scored as correct if they were correct in relation to the new number. The order of task presentation was randomised across participants, but remained the same for the individual participant for each of their testing visits.

During the serial subtraction tasks, participants also completed a computerised tracking task, in which they were required to use the mouse to move a cursor to attempt to track an asterisk that followed a smooth, random on-screen path, moving at a rate of approximately 6 cm/second. Participants were instructed to keep the cursor as close to the asterisk as possible. The distance between the target and the cursor was computed every 100 ms and the resulting data converted to a ‘tracking cost’ score in pixels (with approximately 2.8 pixels/mm for 17-in. monitors) averaged across each minute of task performance.

These tasks were performed in front of a panel of three ‘judges’ who maintained a neutral demeanour throughout the assessment. The chair of the observation panel timed the tasks and provided instructions (i.e. starting number and number to subtract). All panel members made occasional notes. The computer screen, showing the tracking task, was projected onto a screen to give the impression that the panel was closely monitoring progress. The task was recorded using a Dictaphone. Before and immediately after completing the OMS, mood was assessed with the STAI (state) and computer delivered visual analogue scales (VAMS) indicating the participants current level of stress, anxiety, relaxation and calmness. These measures of mood were also repeated every 15 minutes (VAS) or 30 minutes (STAI-state) after completion of the stressor, as per Figure 2 in the main text.

**References**

Kennedy DO, Little W and Scholey AB. (2004) Anxiolytic effects of a combination of Melissa officinalis and Valerlana officinalis during laboratory induced stress. *Journal of Psychopharmacology* 18: A45-A45.

Kirschbaum C, Pirke KM and Hellhammer DH. (1993) The 'Trier Social Stress Test'--a tool for investigating psychobiological stress responses in a laboratory setting. *Neuropsychobiology* 28: 76-81.
