# Supplementary material for: The chronic effects of a combination of herbal extracts (Euphytose®) on psychological mood state and response to a laboratory stressor: A randomised, placebo-controlled, double blind study in healthy humans
Source: J Psychopharmacol. 2022 Jul 23;36(11):1243–56. doi: 10.1177/02698811221112933 (PMC9643820; doi:10.1177/02698811221112933)
Supplement: sj-docx-5-jop-10.1177_02698811221112933 – Supplemental material for The chronic effects of a combination of herbal extracts (Euphytose®) on psychological mood state and response to a laboratory stressor: A randomised, placebo-controlled, double blind study in healthy humans [file sj-docx-5-jop-10.1177_02698811221112933.docx]

**Supplemental file 4 – Cognim^app^ smartphone measures**

### *Mood.* VAMS and Perceived Stress Scale were completed as described previously.

### *Karolinska sleepiness scale (KSS).* The KSS is a 9-point Likert scale designed to measure subjective drowsiness in the preceding 5 minutes to taking the questionnaire. The KSS is sensitive to time of day and time on task effects on subjective drowsiness. Driving performance and EEG indictors of sleep are also highly associated with KSS scores. The KSS is particularly suited to field studies and only takes less than a minute to complete.

### *Simple reaction time.* An upwards pointing arrow was displayed on the screen 30 times with a randomly varying inter-stimulus interval of between 1 and 3 seconds. Participants responded with a single button press as quickly as they could as soon as they saw the arrow appear. The task was scored for mean reaction time (msec).

### *Digit vigilance.* A fixed number appeared on the right of the screen and a series of changing numbers appear on the left side of the screen. Participants were required to make a response when the number on the left matched the number on the right. Task outcomes were accuracy (%), reaction time to correct responses (msec) and number of false alarms. This timed task lasted three minutes.

### *Stroop.* In this computerised version of the classic task 40 words describing one of four colours (‘RED’, ‘YELLOW’, ‘GREEN’, ‘BLUE’) were presented in different coloured fonts in the centre of a computer screen. The participant pressed one of four coloured response buttons in order to identify the font colour (e.g. if the word ‘GREEN’ was presented in a blue font, the correct response would be to respond with the blue button). The presented words were either ‘congruent’ (word and font are the same colour) or ‘incongruent’ (word and font are different colours) and were presented in a random order. The task was scored for number of correct responses overall and reaction time for correct responses overall (msec), and for accuracy (% correct) and reaction time for correct responses for ‘congruent’ and ‘incongruent’ words.

### *Rapid visual information processing (RVIP).* Participants monitored a continuous series of digits for targets of three consecutive odd or three consecutive even digits. The digits were presented at the rate of 100 per minute and the participant responds to the detection of a target string by pressing a response key as quickly as possible. The task was continuous and lasted for three minutes, with 8 correct target strings being presented in each minute. The task was scored for accuracy (%), mean reaction time for correct detections (msec), and false alarms (number).

### *Peg and ball.* Two configurations were shown on the screen. In each there were three coloured balls (blue, green, red) on one of three pegs. The configuration at the top of the screen is the goal configuration and participants must arrange the balls on the starting configuration (shown in the centre of the screen) to match the position of balls in the goal configuration. They were required do this in the least number of moves possible. Participants were required to complete levels 3 to 5 and there are 5 trials within each level. This task was scored for average thinking time (msec), average completion time (msec) and errors (total number of moves in excess of minimum required to complete all trials).
