# Supplementary material for: The chronic effects of a combination of herbal extracts (Euphytose®) on psychological mood state and response to a laboratory stressor: A randomised, placebo-controlled, double blind study in healthy humans
Source: J Psychopharmacol. 2022 Jul 23;36(11):1243–56. doi: 10.1177/02698811221112933 (PMC9643820; doi:10.1177/02698811221112933)
Supplement: sj-docx-6-jop-10.1177_02698811221112933 – Supplemental material for The chronic effects of a combination of herbal extracts (Euphytose®) on psychological mood state and response to a laboratory stressor: A randomised, placebo-controlled, double blind study in healthy humans [file sj-docx-6-jop-10.1177_02698811221112933.docx]

**Supplemental file 5 – Full procedure**

Participants attended the Brain, Performance, Nutrition Research Centre laboratory (Northumbria University, UK) on five separate occasions. The first was an introductory visit and comprised briefing on requirements of the study, obtaining informed written consent, self-report health screening (including blood pressure measurements) and collection of demographic data. Participants were then trained on the cognitive and mood measures that were to be assessed over the course of the study. Participants were provided with a food diary to record what food and drink they had consumed prior to attending their first testing visit, so that it could be replicated prior to each subsequent visit. They were also provided with a stool sampling kit, to bring completed to their first testing visit (to be provided within 24 hours of assessment and stored in the fridge or a cool place). Results from this exploratory outcome are to be reported elsewhere.

Following the introductory visit, participants attended the laboratory at a pre-arranged time in the afternoon between 12.20 pm – 14.10 pm on four separate occasions (visits 1 – 4). The first and third visits comprised the baseline assessments, with visit 1 at least 8 days (but within 28 days) of their introductory session. At visit 1 participants were randomly allocated to the counterbalancing schedule. Visits 2 and 4 were chronic assessments and occurred 15 days (+/- 3 days) after visits 1 and 3, respectively. Between visits 2 and 3 there was a 28-day washout period. Thereafter each visit was identical, except for the intervention consumed between visits 1 and 2 and visits 3 and 4. See **Figure 1** in main text for a schematic depicting the timeline of study.

Prior to each testing visit, participants were asked to avoid alcohol and the intake of analgesic and other over the counter (OTC) medication for 24 hours and from systemic antihistamines for 48 hours. They were allowed to consume their usual caffeinated beverages and breakfast and lunch at home on the day of testing, however all food/beverages apart from those containing caffeine were to be finished at least 1 hour prior to the session and all caffeine containing foods/beverages were to be finished at least 2 hours prior to the session. The food and beverages consumed prior to testing visit 1 were to be replicated prior to all testing visits (the food diary was used to record this). Each visit comprised of an individual screening session where verbal confirmation was obtained that the participant still complied with the inclusion/exclusion criteria and any changes to medications or the participants lifestyle habits were reported. Participants also confirmed that they had not experienced any significant life events since their previous visit.

Following screening, participants provided 5 minute baseline GSR and HR readings and a baseline saliva sample. Questionnaires were completed to assess psychological mood/state including (and in the following order) the STAI-State (completed at least 30 minutes prior to OMS), STAI-Trait, GHQ-12, PSS, POMS and VAMS. After a short (~15 minute) break, participants were taken to an ‘interview’ room where they underwent the OMS for 15 minutes in front of a panel of two observers whilst also being video recorded and having their GSR and HR readings measured throughout. The, STAI-State, and VAMS were completed in the lab immediately prior to and after the OMS and at 30, 60 and 90 minutes post-OMS. Seven saliva samples were collected in total, the first sample was taken at baseline (-45 minutes pre-OMS), the second prior to completion of the OMS (-15 minutes pre-OMS), the third immediately after (0 minutes) and then at 15, 30, 60 and 90 minutes post-OMS (see **Figure 2**). To avoid the potentially confounding effects of the cortisol awakening response on the salivary measures, the testing visits and OMS assessment took place in the afternoon.

Before leaving on testing visits 1 and 3, participants were provided with their treatment and their treatment diary, which was used to record date and time treatment was taken and any illness/medication required over the treatment period. Participants were instructed to take the first dose (2 tablets) that day immediately upon leaving the lab and their second dose (2 tablets) that evening. For the intervening days, participants were advised to take their treatment at mealtimes (2 tablets at breakfast, lunch, dinner), preferably with water or a hot drink. If participants forgot to take a dose, they were advised to note this dose as missed on their diary and continue with the dosing schedule as normal. Missed doses were not replaced. At visits 2 and 4 participants returned any unused treatment and their treatment diaries and consumed their final (breakfast) dose of treatment (2 tablets) at home, 2 hours (minimum 1 hour) prior to attending their session. A kit in order to collect their stool sample prior to attending visit 2 was also provided at the end of testing visit 1. At the end of visit 4 participants completed a treatment guess, were debriefed and remunerated for their time. The testing visits lasted approximatley 2 hours 30 minutes. See **Figure 2** in main text for a schematic depicting the procedure during testing visits 1-4. In order to evaluate the on-going effects of treatment on subjective stress and any potential sedative effects of the intervention, participants were also instructed to complete the Cognim^app^ assessment battery just before breakfast and after lunch on days 7 and 14 in each treatment period following their baseline Cognim^app^ assessments on Day -7 and Day 36. During treatment periods, on days Cognim^app^ assessments were to be completed, subjects were advised to take their treatment with breakfast after participants had completed the pre-breakfast Cognim^app^ assessment and with lunch at least 1 hour (ideally 2 hours) prior to the post-lunch Cognim^app^ assessment. Reminders were sent to participants to ensure this was completed. See **Figure 1** in main text for schematic depicting the study timeline (which also comprises the Cognim^app^ assessments). Given the potential for the MHEP to exert sedative effects, cognitive performance, mood and sleepiness were assessed in the morning and afternoon in the intervening weeks following 7 and 14 days’ of treatment.
